# The Interplay between Tubulins and P450 Cytochromes during *Plasmodium berghei* Invasion of *Anopheles gambiae* Midgut

**DOI:** 10.1371/journal.pone.0024181

**Published:** 2011-08-30

**Authors:** Rute C. Félix, Henrique Silveira

**Affiliations:** UEI Parasitologia Médica, Centro de Malária e Outras Doenças Tropicais, Instituto de Higiene e Medicina Tropical, Universidade Nova de Lisboa, Lisboa, Portugal; Université Pierre et Marie Curie, France

## Abstract

**Background:**

*Plasmodium* infection increases the oxidative stress inside the mosquito, leading to a significant alteration on transcription of *Anopheles gambiae* detoxification genes. Among these detoxification genes several P450 cytochromes and tubulins were differently expressed, suggesting their involvement in the mosquito's response to parasite invasion. P450 cytochromes are usually involved in the metabolism and detoxification of several compounds, but are also regulated by several pathogens, including malaria parasite. Tubulins are extremely important as components of the cytoskeleton, which rearrangement functions as a response to malaria parasite invasion.

**Methodology/Principal Findings:**

Gene silencing methods were used to uncover the effects of *cytochrome P450 reductase*, *tubulinA* and *tubulinB* silencing on the *A. gambiae* response to *Plasmodium berghei* invasion. The role of tubulins in counter infection processes was also investigated by inhibiting their effect. Colchicine, vinblastine and paclitaxel, three different tubulin inhibitors were injected into *A. gambiae* mosquitoes. Twenty-four hours post injection these mosquitoes were infected with *P. berghei* through a blood meal from infected CD1 mice. Cytochrome P450 gene expression was measured using RT-qPCR to detect differences in cytochrome expression between silenced, inhibited and control mosquitoes. Results showed that *cytochrome P450 reductase* silencing, as well as tubulin (*A* and *B*) silencing and inhibition affected the efficiency of *Plasmodium* infection. Silencing and inhibition also affected the expression levels of cytochromes P450.

**Conclusions:**

Our results suggest the existence of a relationship between tubulins and P450 cytochromes during *A. gambiae* immune response to *P. berghei* invasion. One of the P450 cytochromes in this study, *CYP6Z2*, stands out as the potential link in this association. Further work is needed to fully understand the role of tubulin genes in the response to *Plasmodium* infection.

## Introduction

*Plasmodium* infection starts with the ingestion of an infective blood meal. That, together with the parasite invasion of the midgut epithelium promotes an increase of the oxidative stress inside the mosquito, leading to a significant alteration on transcription of *A. gambiae* detoxification genes [1]. Among these, a high number of P450 cytochromes are differently expressed during *Plasmodium* infection, suggesting that they are involved in the mosquito response to parasite invasion.

Insect P450 cytochromes constitute a diverse superfamily of heme-containing enzymes [2] much less studied than P450 cytochromes from mammals and plants, which have well identified and characterized functions [3], [4]. P450 are known to be involved in the metabolism, development and detoxification [5]. They metabolize endogenous compounds like steroids and lipids and exogenous compounds like insecticides [2], [5]. In *A. gambiae* cytochromes from the *CYP6* family have been involved in metabolic resistance to insecticides [6]–[10]. There is also evidence that the transcription of these genes are regulated by the presence of several pathogens, including malaria parasites in the mosquito *A. gambiae* [11], [12].

*Anopheles gambiae* larvae [13] and adults [1] showed P450 cytochromes to be highly enriched in the midgut and in *Drosophila melanogaster* and in *Aedes aegytpi* most P450 cytochromes were also expressed at the midgut [14], [15], suggesting that gut tissue play a major role in xenobiotic detoxification and have a potential role in the protection from injurious exogenous compounds or organisms.

During *Plasmodium* infection transcription alteration of detoxification genes were associated with differential regulation of cytoskeleton genes such as *tubulinA*, *tubulinB* and *actin5C* [1]. In *A. gambiae* microtubules and hence tubulins are of great importance as endothelium cytoskeleton rearrangement may function as a key element during ookinete invasion of the midgut. [16]. A close association between tubulin/microtubules and P450 cytochromes have been described in mammals as disturbance of microtubular dynamics causes a severe impact on the cell viability and function, including the regulation of P450 cytochromes [17]. The microtubule disarray may indirectly change the transcriptional activities of nuclear receptors which are responsible for P450 cytochromes regulation [17]. Furthermore, it was shown that colchicine, an important tubulin inhibitor, down-regulates several P450 cytochromes in human hepatocytes [18]. It was also shown that several tubulin inhibitors are metabolised by P450 cytochromes, so they are likely to induce or repress P450 gene expression [19]. Similarly in *A. gambiae*, regulation of P450 cytochrome expression might be associated with tubulins/microtubules disruption and cause suppression or induction of several P450 cytochromes during the mosquito response to parasite invasion.

The aim of this work was to clarify the role of tubulins in *A. gambiae* during the response to *Plasmodium* infection and its connection with the regulation of an important super-family of detoxification enzymes in *A. gambiae*, the P450 cytochromes.

## Materials and Methods

### Ethics Statement

The maintenance and care of experimental animals was carried out in strict accordance with the recommendations in the Europe Directive 86/609/EEC and Portuguese law (Decreto-Lei 129/92) for biomedical research involving animals. Full details of this study were approved by the Divisão Geral de Veterinária (DGV), Portugal, under Portaria 8 n°1005/92 from 23^rd^ October. All experiments were performed under anesthesia, and all efforts were made to minimize animal suffering. All the authors directly involved with animal manipulation were licensed to conduct research using laboratory animals.

### Mosquitoes

The *A. gambiae* s.s. (molecular M form) of the Yaoundé strain mosquitoes, obtained from Instituto de Higiene e Medicina Tropical (IHMT) *A. gambiae* insectary, were used. The mosquitoes were reared at 26°C and 75% humidity on a 12/12 hour light/dark cycle. Adult mosquitoes were maintained on a 10% glucose solution until blood feeding.

### dsRNA synthesis

Primers were designed to include a T7 promoter sequence plus 20 base pairs (bp) of the sequence of the genes of interest. *Cytochrome P450 reductase* (*CPR*) (Vectorbase: AGAP000500), *tubulinA* (*tubA*) (Vectorbase: AGAP001219) and *tubulinB* (t*ubB*) (Vectorbase: AGAP010510) sequences were used to amplify PCR products using *A. gambiae* genomic DNA as template. An exogenous gene, mouse *beta-2microglobulin* (*B2M*) (GenBank: NM_009735), was used to produce control dsRNA. As described above, a pair of primers that included a T7 promoter sequence plus 22 bp of *B2M* sequence were used to amplify a product using cDNA from *Mus musculus* as template. The gene-specific primers for all the genes are provided in Table S1. Each PCR product was purified using a gel extraction kit (Qiagen) and 1–2 µg of the products were used as template to synthesize dsRNA by in vitro transcription using the MEGAscript T7 kit (Ambion) following the instructions of the manufacturer. dsRNA concentration and quality were assessed by spectrometry and agarose gel.

### Silencing genes

Three day-old female mosquitoes were cold-anaesthetized and injected intrathoraxically with 69 *n*l of 3 µg *µ*l^-1^ solution of dsRNA (207 *n*g) for each gene of interest. In each experiment a control group was injected with dsB2M to serve as reference for intensity of infection and for quantification of gene expression levels. For double-silencing experiments the control group was injected with 138 *n*l of 3 µg *µ*l^−1^ of dsB2M and the test group was injected two times with 138 *n*l of a 1∶1 mix with dstubA and dstubB (3 µg µl^−1^). All the injections were performed using a microinjection system (Nanoject; Drummond Scientific). Gene silencing was confirmed 4 days after dsRNA injection by RT-qPCR using the ribosomal S7 gene (Vectorbase: AGAP010592) for normalisation. Four days after dsRNA injection, female mosquitoes were allowed to feed on *P.berghei* infected mice as described below.

### Tubulin inhibitors injection of mosquitoes

Sugar-fed two to three-day-old female mosquitoes were injected with tubulin inhibitors as described above using a microinjection system. Mosquitoes were injected with 69 *n*l of each inhibitor with final concentration being 1 µM for colchicine and 40 µM for vinblastine and paclitaxel (all inhibitors were from Sigma-Aldrich). Water was used as control for injections with colchicine and vinblastine and water with 1.7% DMSO was used as control for injection with paclitaxel. Twenty-four hours after inhibitors injection female mosquitoes were allowed to feed on *P. berghei* infected mice as described below.

### *Plasmodium berghei* infection of mosquitoes

Female CD1 mice (*Mus musculus*), obtained from the IHMT Animal facility, were intraperitoneally inoculated with 10^7^ *P. berghei* GFP CON parasitised red blood cells. The levels of parasitaemia were measured from blood samples of the mouse tail using Giemsa-stained blood films. When the parasitaemia reached 10–20% and exflagellation was observed, mice were used to infect mosquitoes. Female mosquitoes were allowed to feed directly on *P. berghei* infected mice for up to 30–45 minutes, with regular monitoring to verify that mice were anesthetised. Unfed females were removed from the cage. Fully engorged mosquitoes were kept at 19–21°C and 80% humidity for *P. berghei* development.

### Tissue collection

For mosquitoes with silenced genes, mosquito midguts were collected from pools of 30 mosquitoes 4 days after the silencing and immediately before the blood meal. Tissues were dissected from mosquitoes submerged in ice-cold DEPC treated phosphate-buffered saline (PBS) and transferred to ice-cold RNAlater (Ambion). After incubation at 4°C over night any excess RNAlater was removed and samples were stored at −20°C until RNA extraction. For all groups, mosquito midguts were collected 24 hours post-infection to determine the levels of expression of the genes in study. Eight or nine days post-infection mosquito midguts were also collected to determine infection rate (number of infected mosquitoes over total number of mosquitoes observed) and infection intensity (mean number of oocysts per infected mosquito) by fluorescence. The distribution of parasite numbers in individual mosquitoes between control and experimental groups was compared using the *Mann-Whitney* (*MW*) test. Three independent biological replicas of each experiment were performed.

### Quantitation of gene expression

Total RNA was prepared using the Nucleospin RNAII kit (Macherey-Nagel) according to the manufacturer's instructions. First strand cDNA was synthesized using oligo dT (Roche) and M-MLV Reverse Transcriptase (Promega) as described by the manufacturer. Gene expression was assessed by quantitative real-time PCR with the iQ^TM^ SYBR® Green supermix (Bio-Rad) using the iCycler iQ^TM^ (Bio-Rad). PCR involved an initial denaturation at 95°C for 10 min, 40 cycles of 10 sec at 95°C and 45 sec at 62°C. Fluorescence readings were taken at 62°C after each cycle and a melting curve was obtained (60°C–99°C) to confirm the identity of the PCR product. RT-qPCR measurements were made in triplicate. For gene silencing confirmation the primers used for qPCR amplify a gene fragment non-overlapping the fragment used for dsRNA. Alongside gene silencing confirmation we also measured the levels of expression of several P450 cytochromes in order to check if the different gene silencing and the tubulin inhibition would affect the transcription these genes. Relative quantification results were normalised with the gene that codes for the ribosomal protein *S7* and analysed by the standard curve method, as optimized previously in our lab. Primers used are provided in Table S1. Three independent experiments with three replicates were performed.

### Statistical analysis

For data not normally distributed (oocyst densities) two-sample comparisons were done using a non-parametric test, the *Mann-Withney* (*MW*) test (Graphpad, Prism 5.00). The differences in the infection rate between the control group and the test groups were compared using the *Fisher*'*s Exact test* (*F*) one-tailed (GraphPad, Prism 5.00). Comparisons of mRNA expression levels between the control groups and the test groups were done using the *Mann-Whitney* test one-tailed (GraphPad, prism 5.00).

## Results

### Effect of Silencing CPR in *P. berghei* infection

There are approximately one hundred of highly similar P450 cytochromes in the *A. gambiae* genome and their function tend to be redundant. Therefore silencing each cytochrome individually was not feasible. As an alternative to reduce the activity of P450 cytochromes, the *CPR* gene was silenced, since it is the main electron donor for P450 cytochromes.

**Figure 1:**
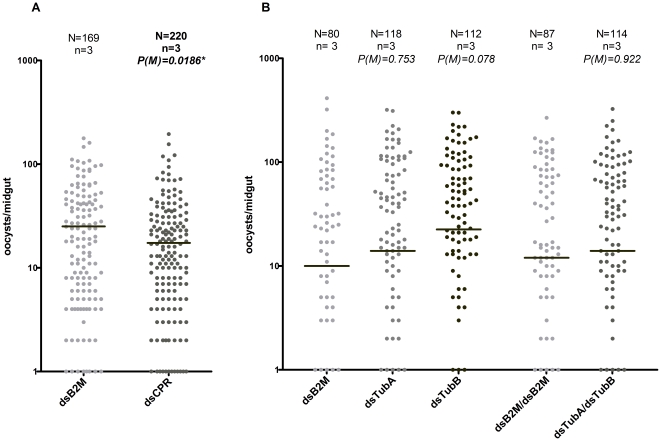
Effect of silencing *CPR* (A), *tubA*, *tubB*, or co-silencing *tubA* and *tubB* (B) on *P. berghei* infection at 8 days after an infected blood meal. The dots represent the number of parasites present on individual midguts, and the median number of oocysts is indicated by the horizontal line, where N is the total number of mosquitoes analyzed and n is the number of independent experiments. Oocysts distributions are compared using *Mann-Whitney* (M) test. * indicates significant differences (*p*<0.05).

Consistent silencing of *CPR* expression (mean = 78%, Table 1) was observed in all experiments, which allowed further analysis of the *in vivo* effects of the reduction of *CPR* activity. In all experiments dsCPR mosquitoes showed a consistent reduction in the infection rate relative to dsB2M mosquitoes, and was significant when all experiments were pooled (*p* = 0,0391, *Fisher*'*s exact* test) (Table 1). When the distributions of oocysts number by infected midgut were compared between the two groups a significant reduction of *P. berghei* infection intensity was observed (*p* = 0.0186, *Mann-Whitney* test) (Figure 1A).

**Table 1:** Effect of silencing *CPR*, *tubA*, *tubB* or co-silencing *tubA* and *tubB* and effect of injecting tubulins inhibitors on *P. berghei* infection in the mosquito.

	N	KD (%)	Infection rate (%)	*P* *(Fisher's Exact test)*	Oocysts range
**Silencing**					
** dsB2M**	169		80.5		0–178
** dsCPR**	220	78	72.3	0.0391*	0.195
** dsB2M**	80		71.3		0–414
** dsTubA**	118	77	70.3	0.5100	0–320
** dsTubB**	112	88.7	76.8	0.2415	0–301
** dsB2M/dsB2M**	36		77.8		0–268
** dsTubA/dsTubB**	69	82.8/83.8	66.7	0.1690	0–161
**Chemical inhibition**					
** Control**	138	n/a	69.6		0–450
** Paclitaxel 40** µ**M**	97	n/a	88.7	0.0004***	0–359
** Control**	163	n/a	82.2		0–305
** Vinblastine 40** µ**M**	178	n/a	82.6	0.1214	0–250

N – total number of mosquitoes; Knock down (KD)(%) - 100*((mean expression dsB2M – mean expression dsCPR, dsTubA or dsTubB)/mean expression dsB2M); Infection rate (%) – 100*(n° of infected mosquitoes/total number of mosquitoes dissected); *Fisher's exact test* to analyse the differences in the infection rate between the control group and the test group; *indicates significant differences (*p*<0.05); *** indicates significant differences (*p*<0.001).

### Effect of tubulins silencing in *P. berghei* infection

*TubulinA* and *tubulinB* were already reported as differentially expressed during *Plasmodium* infection [1], [11], [13], [16], [20]. These tubulins are members of the microtubules that constitute the cytoskeleton. In the mosquito, the ingestion of a blood meal causes dramatic morphological changes in the cytoskeleton and their components [21]. This cytoskeleton rearrangement is seen as a robust molecular response to ookinete invasion [16]. Thus, each tubulin individually or both at the same time were silenced to determine the effect of the absence of tubulins on *Plasmodium* infection.

High levels of silenced tubulin expression were obtained both for the single silencing (*tubA* mean = 77.0% and *tubB* mean = 88.7%) and for the double silencing (*tubA* mean = 82.8% and *tubB* mean = 83.8%) (Table 1) When *tubB* was single silenced a slightly higher infection rate was observed, but this rate was essentially similar between the tubulins single or co-silenced and the dsB2M mosquitoes.

The distribution of oocysts number by infected midgut, in *tubA* and *tubB* single silencing, showed consistently higher infection intensity than the control groups, although this difference was not significant (Figure 1B). In the co-silencing, the infection intensity was similar between the dstubA/dstubB mosquitoes and the control group (Figure 1B).

### Effect of tubulins inhibitors injection in *P. berghei* infection

**Figure 2:**
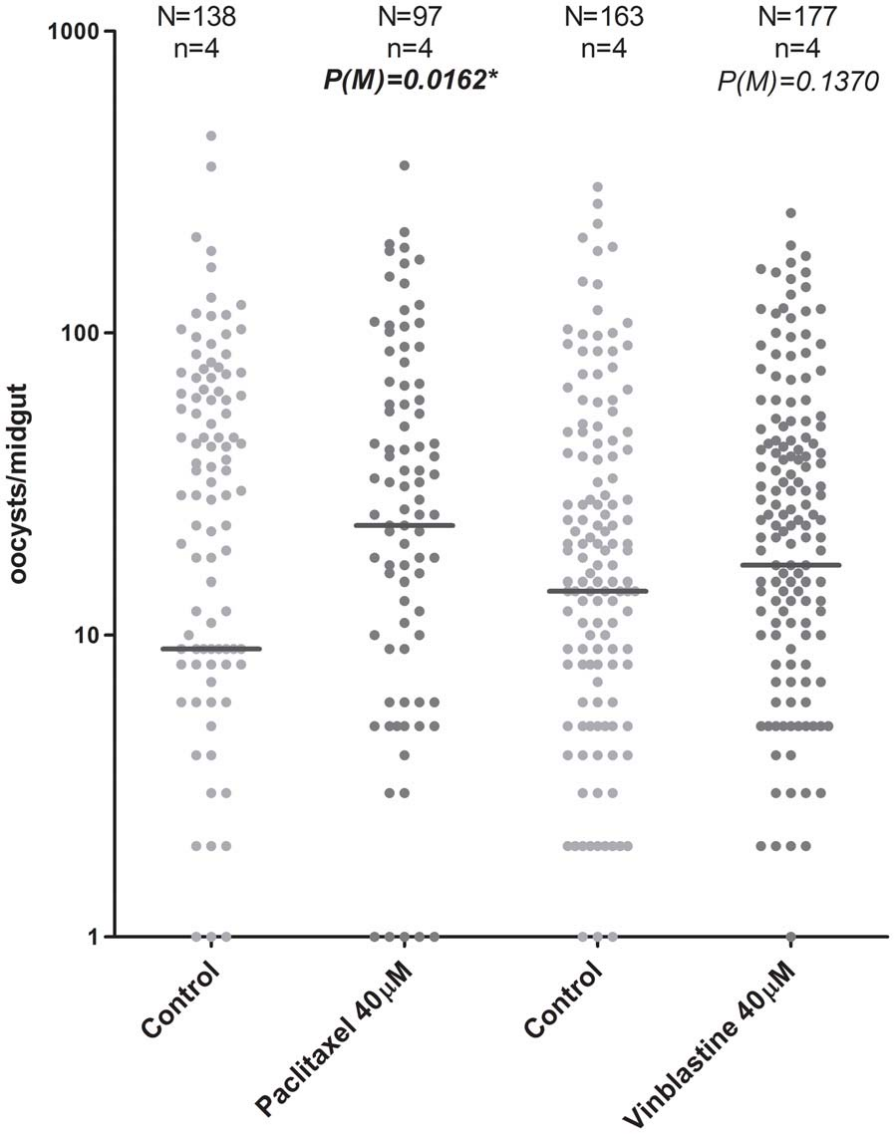
Effect of tubulin inhibitors, paclitaxel and vinblastine, on *P. berghei* infection at 8 days after an infected blood meal. The dots represent the number of parasites present on individual midguts and the median number of oocysts is represented by a horizontal line. Vinblastine 40 µM, paclitaxel 40 µM. N is the total number of mosquitoes analyzed and n is the number of independent experiments. Oocysts distributions were compared using *Mann-Whitney* (M) test. *indicates significant differences (*p*<0.05)

Mosquitoes were injected with 3 tubulin inhibitors, one of each of the three major classes of tubulin inhibitors. A consistent increase in the infection rate of mosquitoes treated with paclitaxel, from the class taxoids, was observed in the 3 experiments when compared with to the control mosquitoes. This increase was highly significant (*p* = 0.0004, *Fisher's exact* test) (Table 1). The number of oocysts by infected midgut was significantly different between the control group and the group treated with paclitaxel (*p* = 0.0162, *Mann-Whitney* test) (Figure 2).

When mosquitoes were treated with vinblastine, from the class ‘*Vinca*’ alkaloids, there were no significant differences between the control group and the test group concerning either the infection rate or the distribution of oocysts by infected midgut (Figure 2, Table 1).

When mosquitoes were injected with colchicine, from the class of colchicine binders, a high mortality was observed, although 24 h after the blood meal we were able to collect enough midguts to analyse by semi quantitative RT-PCR. The remaining mosquitoes did not survive and were all dead at day 3 after the blood meal, therefore the number of oocysts by infected midgut was not possible to determine. So, for this treatment only RT-PCR data was analysed.

### Effect of CPR silencing in P450 cytochromes expression

**Figure 3:**
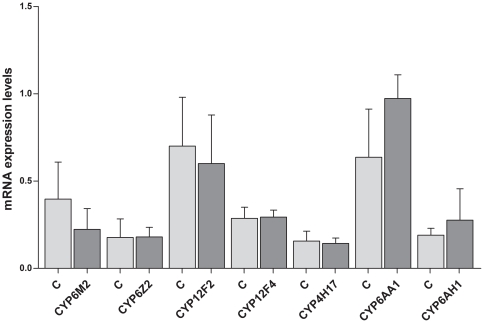
P450 cytochromes mRNA expression levels in control (dsB2M-injected) and *CPR* silenced (dsCPR-injected) midguts. Midguts were collected 24 hours after a *P. berghei* infected blood meal. Data are shown as mean ± SEM.

As *CPR* inhibition eliminates all microsomal P450 activity in the mouse model [22], the same approach was applied to evaluate whether P450 cytochromes have some role in controlling *Plasmodium* infection. The expression levels of seven P450 cytochromes, chosen among the ones reported as differentially expressed during *Plasmodium* infection [1], were analysed and differences were observed when gene expression levels were compared between the control group and the silenced *CPR* group (Figure 3). *CYP6M2* (Vectorbase: AGAP008212) and *CYP6AA1* (Vectorbase: AGAP007480) showed more pronounced differences between the control group and silenced *CPR* group, even so they were not significant (Figure 3, *Mann-Whitney test* one-tailed).

### Effect of tubulins silencing in P450 cytochromes expression

**Figure 4:**
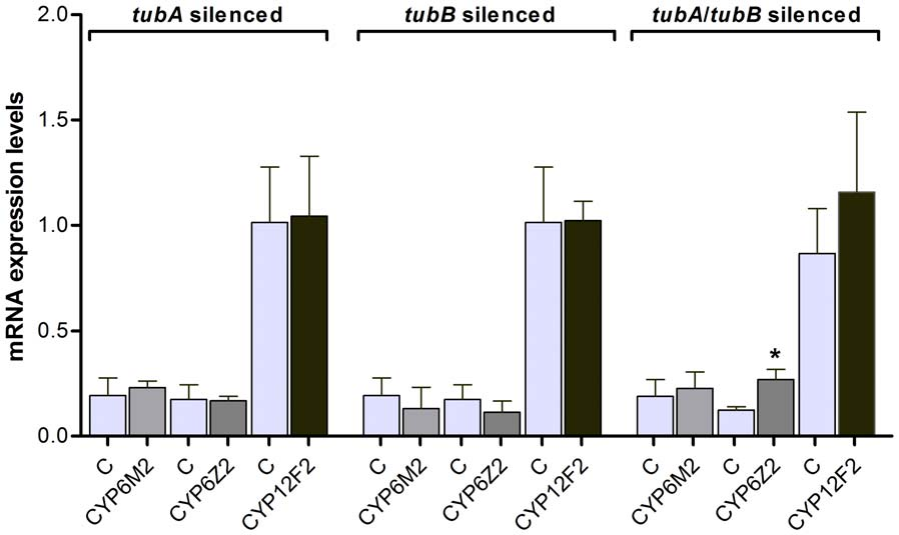
Effect of silencing *tubA* or *tubB* (or co-silencing *tubA* and *tubB*) on P450 cytochromes mRNA expression levels in midguts. Midguts were collected 24 hours after a *P. berghei* infected blood meal. Data are shown as mean ± SEM. *indicates significant differences (*p*<0.05) by *Mann-Whitney* test one-tailed.

The expression of three P450 cytochromes (*CYP6M2*, *CYP6Z2* (Vectorbase: AGAP008018), and *CYP12F2* (Vectorbase: AGAP008021)), already reported as differentially expressed upon *Plasmodium* infection [1], [23] and associated with insecticide resistance [7], [9], was analyzed in order to detect the effect of microtubule disruption on P450 cytochromes. When *tubA* and *tubB* were silenced individually no significant differences in expression were observed. Even so, when just *tubB* was silenced, slight decrease in expression was observed for *CYP6M2* and *CYP6Z2,* while *CYP12F2* had an opposite behavior. However, when both tubulins were KD simultaneously an increased expression was observed in all P450 cytochromes analyzed, being the difference observed in *CYP6Z2* statistically significant (Figure 4).

### Effect of tubulins inhibitors injection in P450 cytochromes expression

**Figure 5:**
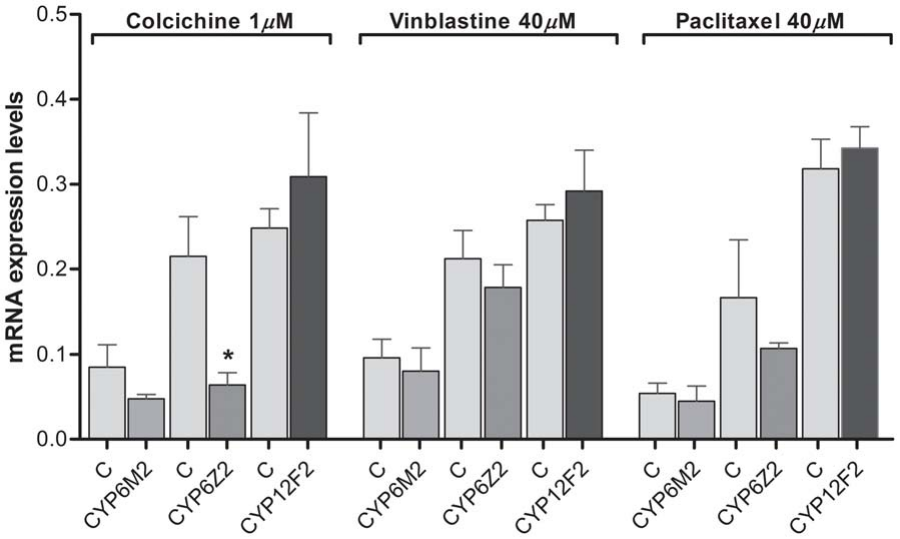
Effect of tubulin inhibitors in P450 cytochromes mRNA expression levels in midguts. Midguts were collected 24 hours after a *P. berghei* infected blood meal. Colcichine 1 µM, vinblastine 40 µM and paclitaxel 40 µM. Data are shown as mean ± SEM. *indicates significant differences (*p*<0.05) by *Mann-Whitney* test one-tailed.

The effect of tubulin inhibitors (colchicine, vinblastine and paclitaxel) was analyzed on three P450 cytochromes (*CYP6M2, CYP6Z2* and *CYP12F2*). We observed that, as with the silencing experiments, *CYP12F2* had always a different behavior from the other two P450 cytochromes studied (Figure 5). *CYP12F2* expression levels were up-regulated after treatment with all the tubulin inhibitors, the exact opposite was observed for *CYP6M2* and *CYP6Z2,* which were down-regulated with all the tubulin inhibitors. Colchicine was the tubulin inhibitor that caused a higher response from all the genes in the study and *CYP6Z2* was the gene with the highest expression levels changes observed (Figure 5). Furthermore, *CYP6Z2* was the only P450 cytochrome where the differences between the control group and the tubulin inhibitor injected group were statistically significant (Figure 5, *p* = 0.05, *Mann-Whitney test* one-tailed) for the colchicine experiment.

## Discussion

Silencing the *CPR* gene showed that parasites become less effective in the invasion of midgut epithelium with this gene silenced, as proven by the significant reduction of the infection rate and the intensity of infection. However, the reason why this happens is still unknown. On the other hand, although being the main electron donor for P450 cytochromes activity, significant differences were not found in P450 expression profiles when *CPR*-silenced mosquitoes *versus* non-silenced ones were compared. One hypothesis is that P450 cytochromes could receive electrons from other donors and that may be the reason why no differences were observed. For example, microsomal P450 cytochromes can receive electrons from cytochrome *b_5_* and cytochrome *b_5_* reductase, while P450 cytochromes in mitochondrial systems can receive electrons from an adrenodoxin-like ferredoxin coupled to an adrenodoxin reductase [2]. Also, regulation of P450 cytochrome expression depends of nuclear receptors, which may be affected by multiple mechanisms, so the silencing of the *CPR* might not have a direct impact on P450 transcription, which might explain why transcription of the P450 cytochromes studied was not affected. Knowing that *CPR* silencing did not affect P450 cytochrome expression, the reduction of *Plasmodium* infection rate and intensity observed with the silencing of this gene was not associated with P450 cytochromes being unable to perform their functions, as their expression levels were unchanged.

Tubulins are important members of microtubules that constitute the cytoskeleton. Microtubules are essential in cell division, contribute to the maintenance of cell shape and integrity and play a major role in cell motility among other important functions [24]. Their most significant characteristic is the ability to polymerize (assemble) and depolymerise (disassemble) reversibly, depending on local conditions [24]. Cytoskeleton rearrangement functions as a response to *Plasmodium* infection [16], and an association between microtubules and P450 cytochromes has been reported [17], [18]. Both tubulin genes and many P450 cytochromes were differentially expressed during *Plasmodium* infection [1], so a connection between tubulins and P450 cytochromes in response to *Plasmodium* infection in *A. gambiae* was suggested. Silencing *tubA*, *tubB* and co-silencing *tubA*, *tubB* was performed and although some differences in the *Plasmodium* infection rate and intensity between the tested groups were observed, significant changes were not observed in infection rate neither with the single silencing nor the co-silencing. One possibility is that the cytoskeleton rearrangement is extremely complex, involving a large array of genes, and that tubulins are not crucial to the rearrangement resulting from the response to *Plasmodium*. Another possibility is that this method was not capable of truly silencing tubulins, as microtubules are dynamic polymers that are continuously being built and degraded, even if they were effectively silenced they would rapidly recover and thus mask the silencing effect. Even so, this microtubules turnover is thought to play a major role in several cellular processes requiring a change in cell shape [25], which may include the cytoskeleton rearrangement which function as a response to *Plasmodium* infection. Although not significant, oocyst density was always higher in tubulin knock down groups suggesting differences in the infection response among groups

Concerning the tubulin silencing effect in the expression of P450 cytochromes, *CYP12F2* had always a different behavior compared to the other P450 cytochromes. This may be due to the fact that these cytochromes lie at different locations: *CYP12F2* is a mitochondrial cytochrome while *CYP6M2* and *CYP6Z2* are microsomal cytochromes. In addition, they have different ways to interact with electron donors as well as different electron donors, as said above. Tubulin silencing seemed to have no effect on *CYP12F2* levels of expression, possibly because this P450 cytochrome is located in the mitochondria, thus tubulin silencing would not be able to influence genes within these organelles. An up-regulation of *CYP6M2* and *CYP6Z2* with co-silencing of *tubA* and *tubB* was observed. As said previously, microtubule disarray limits the signaling by nuclear receptors involved in P450 cytochrome regulation in mammals [17], [19], consequently, the differences observed in genes expression levels may be caused by changes in nuclear receptors expression levels in response to the absence of tubulin expression.

Another approach was made to study the role of tubulins in response to *Plasmodium* infection, the injection of tubulins inhibitors. Colchicine is the inhibitor with more toxicity to mammal cells [24], [26], thus it may be also very toxic to mosquitoes and that could be the reason for the high mortality of mosquitoes in these experiments. Paclitaxel injection caused a significant increased in infection rate and oocysts density in the inhibited group, while in the vinblastine injected mosquitoes there was only slightly differences between groups. The different effect of these two inhibitors in the *Plasmodium* infection must be due to the fact that these interact with microtubules via different mechanisms, while vinblastine aggregates tubulin and leads to microtubule depolymerisation, paclitaxel stabilizes microtubules by binding them to a polymer; additionally they have different binding-sites, which influence their role [24], [26]. Nevertheless, these compounds may not inhibit totally tubulin, for instance, with paclitaxel microtubules can still turnover, but not to the same extent as without it [27]. As with the other inhibitors, they could just make microtubules less available, as occurs with paclitaxel [27]. On the other hand, administration of tubulin inhibitors may also be acting in the parasite tubulins in the mosquito midgut, being responsible for changes in the parasitemia levels, however, it is well known that tubulins inhibitors bind tubulins from different species with generally different affinities [24].

The down-regulation of *CYP6M2* and *CYP6Z2* expression levels with all the inhibitors was somewhat expected since microtubules-interfering agents were used, in this case colchicine, vinblastine and paclitaxel, they change the transcriptional activity of nuclear receptors responsible for the regulation of several P450 cytochromes [18], [28]. Furthermore, as these inhibitors are metabolised by P450 cytochromes they could function as inducers or repressors of P450 cytochrome expression [19]. Overall, compared with tubulin silencing, it seems that tubulins inhibition had a higher effect on P450 expression levels, which suggests that different mechanisms of inhibition may affect P450 cytochromes expression in dissimilar ways. Accordingly different *CYP12F2* expression levels were obtain between the methods suggesting that they affect differently the expression of mitochondrial P450 cytochromes.

Colchicine was the inhibitor that caused higher changes in P450 expression levels, which was an expected result, as it was already reported that colchicine down-regulated several P450 cytochromes in mammals [18]. *CYP6Z2* was the P450 cytochrome who showed significant differences in both the co-silencing of *tubA* and *tubB* and the inhibition with colchicine experiments, although in opposite directions. The reason why these two methods, with the same aim, gave such different results is not yet known, but is probably due to the different mechanism of action of the two approaches. Nevertheless, *CYP6Z2* is the most promising candidate to be directly involved with the tubulin/microtubule disarray.

In conclusion, we demonstrated that *CPR* and tubulin silencing and inhibition affected the mosquito's response to *Plasmodium*. We also showed a possible association between tubulins and P450 cytochromes in response to malaria parasite, identifying one P450 cytochrome, *CYP6Z2* as a candidate for this association. Although these silencing and inhibitions did not account for major parasite number losses during *Plasmodium* infection of the midgut they suggest that these genes may be part of a more complex response to parasite invasion. These results corroborate the importance of further studying tubulin genes to fully understand their role in the *Plasmodium* response.

## Supporting Information

Table S1 **Primers used for dsRNA synthesis and semi-quantitative real time PCR experiments and respective product length.** The underlined base pairs are the T7 promoter sequence included in the primers.
(DOC)
