# Supplementary material for: The Interplay between Tubulins and P450 Cytochromes during Plasmodium berghei Invasion of Anopheles gambiae Midgut
Source: PLoS One. 2011 Aug 30;6(8):e24181. doi: 10.1371/journal.pone.0024181 (PMC3166158; doi:10.1371/journal.pone.0024181)
Supplement: Table S1 — Primers used for dsRNA synthesis and semi-quantitative real time PCR experiments and respective product length. The underlined base pairs are the T7 promoter sequence included in the primers. (DOC) [file pone.0024181.s001.doc]

**Table S1**.

| Primer | **Vectorbase**  **(accession number)** | **Sequence (5’ to 3’)** | **Product lenght(bp)** |
| --- | --- | --- | --- |
| dsCPR_Fwd | AGAP000500 | TAATACGACTCACTATAGGGAGATACTGCGGCGAGGAGAAGGAC | 380 |
| dsCPR_Rev |  | TAATACGACTCACTATAGGGAGACGGAACTGGCTCTTGCGGATG |  |
| dsTubA_Fwd | AGAP001219 | TAATACGACTCACTATAGGGAGACAAGGAAGATGCCGCCAAC | 432 |
| dsTubA_Rev |  | TAATACGACTCACTATAGGGAGAGTGATGGACGACACAATCTGG |  |
| dsTubB_Fwd | AGAP010510 | TAATACGACTCACTATAGGGAGAGGTGGAGAACACGGACGAGAC | 498 |
| dsTubB_Rev |  | TAATACGACTCACTATAGGGAGACGGCGGAATATCACAGACGGC |  |
| dsb2m_Fwd | GeneBank: | TAATACGACTCACTATAGGGAGAcacccccactgagactgataca | 447 |
| dsb2m_Rev | NM_009735 | TAATACGACTCACTATAGGGAGAcacccccactgagactgataca |  |
| S7_RT_Fwd | AGAP010592 | GCCATCCTGGAGGATCTGGTA | 132 |
| S7_RT_Rev |  | CGATGGTGGTCTGCTGTTCTTATCC |  |
|  |  |  |  |
| CPR_RT_Fwd | AGAP000500 | CGGTGCTGGTGAAGTACGAGAC | 136 |
| CPR_RT_Rev |  | CGGAACTGGCTCTTGCGGATG |  |
| CYP6M2_RT_Fwd | AGAP008212 | AAGTCGGATGATGATTCGCTAACG | 169 |
| CYP6M2_RT_Rev |  | GCAGGATTTCTCTCACACACTCAC |  |
| CYP6Z2_RT_Fwd | AGAP008218 | CCGTTCGTCTGGTGTATTTATTTGTC | 73 |
| CYP6Z2_RT_Rev |  | CAATTCAGGCTGGAGAGATGTCATG |  |
| CYP12F2_RT_Fwd | AGAP008021 | GCTATGATGGAGTTGGAGATGATTAC | 118 |
| CYP12F2_RT_Rev |  | GCAGCGGATTGGCAGGAATGTTG |  |
| CYP12F4_RT_Fwd | AGAP008018 | CGGTTGGCAATGATGGAGATGGAG | 121 |
| CYP12F4_RT_Rev |  | GATCGTTCGCAGGTATGTTGACAAG |  |
| CYP4H17_RT_Fwd | AGAP008358 | TGGATCTGGTGGTGAAGGAGTC | 106 |
| CYP4H17_RT_Rev |  | GCCTGCTGGAATGGTAGTGCC |  |
| CYP6AA1_RT_Fwd | AGAP002862 | ACTCCACGACGGCAAGATAACG | 99 |
| CYP6AA1_RT_Rev |  | TGCGGAACTGGCGGATACATAC |  |
| CYP6AH1_RT_Fwd | AGAP007480 | TCTCGTCGGCCATTCGGTAACG | 122 |
| CYP6AH1_RT_Rev |  | GCTCCTTCACTACACGGTCCTG |  |
